# Efficacy and safety of antibiotics for treatment of leptospirosis: a systematic review and network meta-analysis

**DOI:** 10.1186/s13643-024-02519-y

**Published:** 2024-04-16

**Authors:** Zhenhua Ji, Miaomiao Jian, Xuan Su, Yingyi Pan, Yi Duan, Weijie Ma, Lei Zhong, Jiaru Yang, Jieqin Song, Xinya Wu, Li Gao, Weijiang Ma, Jing Kong, Bingxue Li, Jinjing Chen, Meixiao Liu, Yuxin Fan, Li Peng, Yan Dong, Fukai Bao, Aihua Liu

**Affiliations:** 1 Evidence-Based Medicine Team, The Institute for Tropical Medicine, Faculty of Basic Medical Science, Kunming Medical University, Kunming, 650500 Yunnan China; 2 The Institute of Oncology, Yunnan Cancer Hospital, Kunming Medical University, Kunming, 650100 Yunnan China; 3 Yunnan Province Key Laboratory of Children’s Major Diseases Research, The Affiliated Children Hospital, Kunming Medical University, Kunming, 650030 Yunnan China; 4 Department of Biochemistry and Molecular Biology, Biomedicine Discovery Institute, Monash University, Melbourne, VIC 3800 Australia

**Keywords:** Leptospirosis, *Leptospira*, Antibiotic, Efficacy, Safety, Network meta-analysis

## Abstract

**Background:**

Leptospirosis, an important zoonotic bacterial disease, commonly affects resource-poor populations and results in significant morbidity and mortality worldwide. The value of antibiotics in leptospirosis remains unclear, as evidenced by the conflicting opinions published.

**Methods:**

We conducted a search in the PubMed, Web of Science, and Cochrane Library databases for studies. These studies included clinical trials and retrospective studies that evaluated the efficacy or safety of antibiotics for leptospirosis treatment. The primary outcomes assessed were defervescence time, mortality rate, and hospital stays. Subgroup analyses were performed based on whether there were cases involving children and whether there were cases of severe jaundice. Safety was defined as the prevalence of adverse events associated with the use of antibiotics. *p* scores were utilized to rank the efficacy of the antibiotics.

**Results:**

There are included 9 randomized controlled trials (RCTs), 1 control trial (CT), and 3 retrospective studies (RS) involving 920 patients and 8 antibiotics. Six antibiotics resulted in significantly shorter defervescence times compared to the control, namely cefotaxime (MD, − 1.88; 95% CI =  − 2.60 to − 1.15), azithromycin (MD, − 1.74; 95% CI =  − 2.52 to − 0.95), doxycycline (MD, − 1.53; 95% CI =  − 2.05 to − 1.00), ceftriaxone (MD, − 1.22; 95% CI =  − 1.89 to − 0.55), penicillin (MD, − 1.22; 95% CI =  − 1.80 to − 0.64), and penicillin or ampicillin (MD, − 0.08; 95% CI =  − 1.01 to − 0.59). The antibiotics were not effective in reducing the mortality and hospital stays. Common adverse reactions to antibiotics included Jarisch–Herxheimer reaction, rash, headache, and digestive reactions (nausea, vomiting, diarrhea, abdominal pain, and others).

**Conclusions:**

Findings recommend that leptospirosis patients be treated with antibiotics, which significantly reduced the leptospirosis defervescence time. Cephalosporins, doxycycline, and penicillin are suggested, and azithromycin may be a suitable alternative for drug-resistant cases.

**Systematic review registration:**

PROSPERO CRD42022354938.

**Supplementary Information:**

The online version contains supplementary material available at 10.1186/s13643-024-02519-y.

## Background

Leptospirosis is an endemic zoonotic infection with significant implications for human health, particularly for agricultural workers and those who engage in outdoor activities in endemic areas. *Leptospira* species are divided into pathogenic and putrefactive spirochetes and comprise more than 250 pathogenic species [1, 2]. These are aerobic spirochetes measuring 6 to 20 μm in length and 0.1 μm in diameter [3]. Leptospirosis is contracted when leptospires enter the body through the skin or mucous membranes of the mouth and conjunctiva. Transmission is either direct, from host to host, or indirect, via soil, infected animal urine, or contaminated water [4]. Incidence rates are underestimated due to a lack of disease awareness and relatively inaccessible and insufficiently rapid diagnostics [5]. Leptospirosis has protean manifestations, often resembling the clinical presentations of other diseases [5]. The disease most commonly affects the kidney and liver [6, 7] and can progress to vasculitis and multi-organ invasion. Most infections are subclinical or mild, with symptoms such as fever, chills, headache, severe myalgia, conjunctival suffusion, anorexia, nausea, vomiting, and prostration usually characterizing acute infections. Leptospirosis usually presents as a non-specific acute febrile illness with similar signs and symptoms to dengue, influenza, and rickettsial infections [8]. Despite its mild initial presentation, delayed intervention leads to severe and possibly fatal Weil’s disease, characterized by hemorrhage, jaundice, renal failure [1], and even nervous system involvement [9]. The clinicopathological features include azotemia, hyperbilirubinemia, elevated liver enzyme levels, and thrombocytopenia [7].

Symptomless *Leptospira* infections are common in endemic areas and affect resource-poor populations in Malaysia, India, Sri Lanka, and Brazil, resulting in significant morbidity and mortality [4]. *Leptospira* are estimated to cause one million infections and approximately 58,900 deaths annually with a case–fatality ratio of 6.85%, yet progress on leptospirosis treatments has been minimal [2]. Livestock and wildlife infections also result in economic livestock industry losses [10]. The main antibiotics currently used to treat leptospirosis include penicillin, ceftriaxone, doxycycline, oxytetracycline, and macrolides (azithromycin or clarithromycin) [5, 11, 12]. Mild cases may not require antibiotics or can be treated with oral doxycycline. More serious cases require intensive care and intravenous penicillin or ceftriaxone [5, 12]. Individuals allergic to penicillin or ceftriaxone may use doxycycline or a macrolide (azithromycin or clarithromycin) [11]. However, despite the ubiquity of leptospirosis with an estimated one million annual global cases, the effectiveness of these antibiotics against leptospirosis is unclear, and treatment choices remain controversial [1, 13–16]. This study aims to discern the best antibiotic options to treat leptospirosis.

The lack of quantitative efficacy comparisons between different antibiotics due to technical limitations has compromised the scope of previous meta-analyses and review articles [13–16]. To address this limitation, we conducted a systematic analysis of data from randomized clinical trials (RCTs), clinical trials (CTs), and retrospective studies (RSs) using network meta-analysis (NMA) to assess the efficacy and safety of antibiotic treatment for leptospirosis. Our aim with this NMA was to compare and rank different antibiotics to evaluate the efficacy and safety of antibiotic therapy for leptospirosis.

## Methods

We developed and followed a protocol for all steps of our systematic review and meta-analysis (PROSPERO CRD42022354938) and reported the study in accordance with the Preferred Reporting Items for Systematic Reviews and Meta-Analysis (PRISMA) guidelines [17] (eTable 1).

### Search strategies and inclusion criteria

We conducted a comprehensive search of the three databases PubMed, Web of Science, and Cochrane Library from the date of creation to December 26, 2023, and the search was completed on January 2, 2024. We conducted three separate searches to maximize data collection, using terms like “leptospirosis,” “antibiotic,” and “controlled trials” (eTable 2).

The eligible studies met the following criteria: (i) controlled or retrospective clinical trials; (ii) laboratory-confirmed leptospirosis diagnoses via microscopic agglutination test (MAT), enzyme-linked immunosorbent assay (ELISA), polymerase chain reaction (PCR), or histopathological evaluation in hospitalized patients; (iii) evaluation of drug efficacy or safety for leptospirosis treatment; and (iv) written in English. We excluded studies on the basis of the following criteria: (i) incomplete data; (ii) review, case report, or comment to editors (lacking primary data); (iii) repeated publication; and (iv) patients unable to complete the therapy.

Two reviewers assessed all included studies independently (JMM and SX). Any disagreements were addressed during discussions with a third reviewer (FB or AL) until a consensus was reached.

### Data extraction and outcomes

After training, two individuals reviewed the abstracts independently and identified articles for detailed assessment. In case of disagreement, the two parties discussed and resolved the issue or referred it to a third researcher for a final decision. Then, they extracted data from each included study and entered the results into a database. Collected data include first author, year of publication, country, screening test used, sample size, antibiotic dose, defervescence time, hospital stays, deaths, and adverse effects. We defined effectiveness on the basis of defervescence time, mortality, and hospital stays (lower values indicate better drug efficacy).

### Quality of evidence and risk of bias

We assessed the risk of bias for each included study using the methodology established by the Cochrane Collaboration [18]. This involved assessing the validity of data from the included studies and assigning a judgement of either “low risk,” “high risk,” or “unclear risk” to each entry in a “risk of bias table.” Entries assigned an “unclear risk” indicate either lack of information or uncertainty over the potential for bias (eTable 3). The extent to which a Cochrane review can draw conclusions regarding the effects of an intervention depends on the validity of the data obtained from the included studies.

### Statistical analysis

We used the Netmeta package of R version 4.2.1 and Cytoscape 3.9.1 to perform this NMA.

Data transformation before analysis was primarily based on the method by Hozo et al. and Cai et al. [19, 20]. Our efficacy assessment employed NMA frequency analysis methods by using the Netmeta package to synthesize total effects, analyze heterogeneity, and calculate rankings. Network plots graphed were by NAM analysis results, Cytoscape graphing (Fig. 2). We selected a fixed-effect or random-effect model based on the heterogeneity to synthesize the study effect sizes. We used *Q* and *I*^2^ tests to evaluate the statistical heterogeneity among studies, with an *I*^2^ > 50% indicating statistically significant heterogeneity. We used odds ratios (ORs) and 95% confidence intervals (95% CIs) to report the effect size for mortality and mean deviations (MDs) and 95% CIs to report the effect size for assessing the defervescence time and hospital stays. Antibiotic effectiveness of different subgroups was finally plotted in Fig. 3.

Inconsistency is a critical indicator for assessing the quality of an NMA (which reflects the difference in the effect estimate between direct and indirect evidence). Hence, we applied the back-calculation method to assess the inconsistency of this NMA. This method is based on the *Z* test and determines the inconsistency by the *p*-value; if *p* < 0.05, it indicates the presence of inconsistency in the NMA [21] (Fig. 4). Next, we ranked the efficacy of drugs according to the NMA results. We used *p* scores to measure the extent of certainty that a treatment was better than others, averaged over all competing therapies [22]. Subsequently, we used the *p* score as a metric to assess the efficacy ranking. *p* scores ranged from 0 (worst) to 1 (best), with higher scores indicating better efficacy. Forest and funnel plots were generated to assess the overall effect size and identify any publication bias. We used Begg’s funnel plot and Egger’s test to detect potential publication bias, with *p* < 0.05 considered as statistically significant [23, 24].

## Results

### Study characteristics

**Fig. 1:**
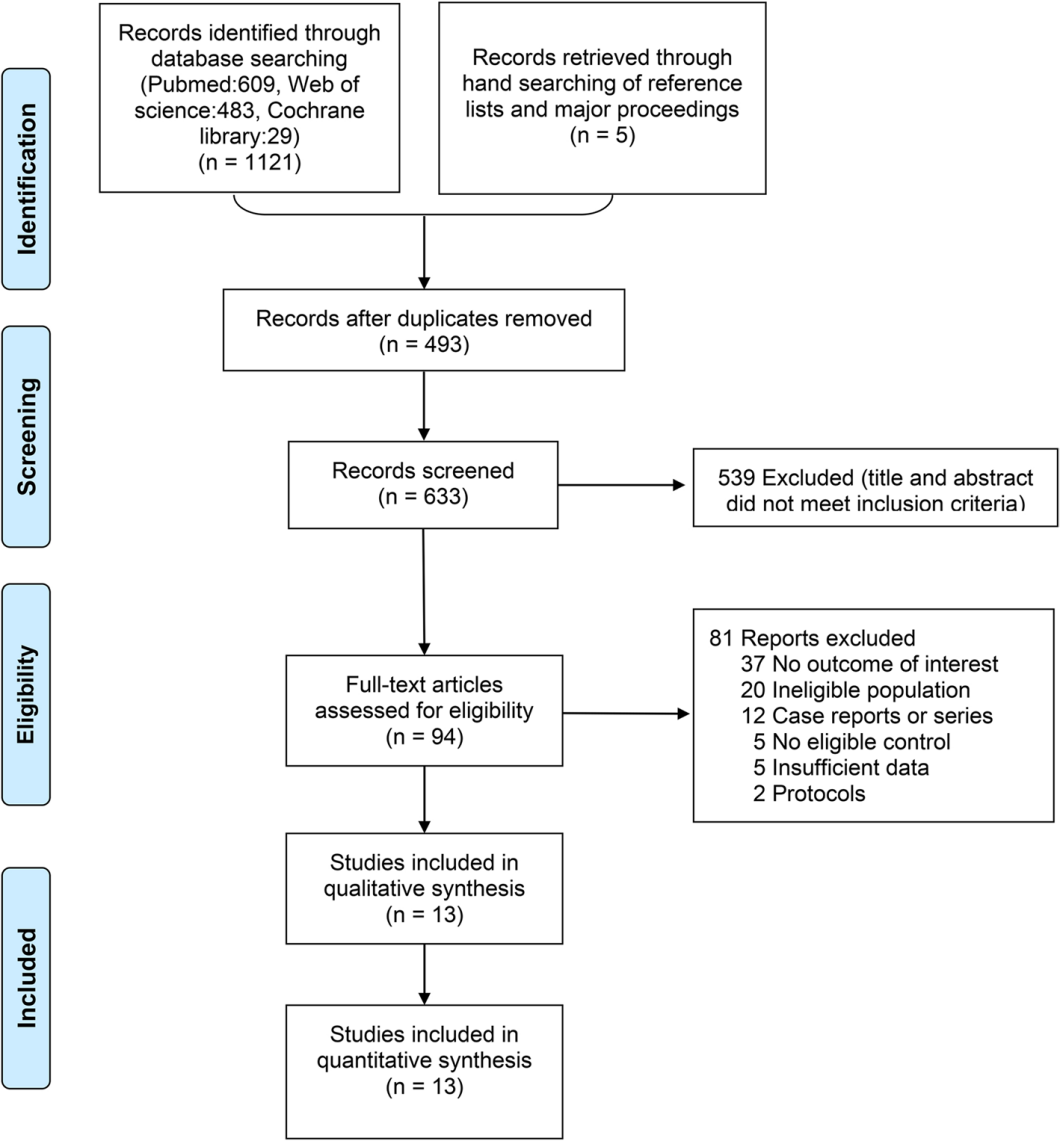
Literature flowchart

We obtained 1126 articles after searching the databases three times (609 papers from PubMed, 483 from Embase, and 29 from the Cochrane Library). The other 5 articles were identified through manual searches. We included 13 eligible articles [25–37] after excluding 493 duplicates and 620 ineligible articles. These included clinical trials were published between 1954 and 2012 and included 920 patients comparing the efficacy and safety of 8 antibiotics. Figure 1 illustrates the study selection process, and Table 1 presents the main characteristics of the included studies. The risk of bias assessment can be found in Supplemental Material 3.

**Table 1:** Studies included in the present review

Study ID	Study types	Diagnostic methods	Therapy duration	Treatment groups	Patients				
					**Source of infection**	**Gender**	**Age**	**Type of leptospirosis**	**Total number**
Doherty RL [25] (1954)	RS	MAT	Unclear	Penicillin low dosage, 100,000 units/3 h; high dosage, 500,000 units/3 h vs*.* chloramphenicol vs. no antibiotic	Australia	Male and female	Children and adults	Unclear	111
Fairburn AC [26] (1956)	CT	MAT	At least 5 days	Penicillin 600,000 units/6 h vs*.* chloramphenicol 0.5 g/6 h vs. no antibiotics	Malaya	Male	Adults	Unclear	83
Russell RW [27] (1958)	RCT	Blood culture or serological tests	At least 5 days	Oxytetracycline 1.5 g followed by 0.5 g/6 h vs*.* placebo	Malaysia	Male and female	Adults	Unclear	52
McClain JB [28] (1984)	RCT	MAT, *Leptospira* cultures	7 days	Doxycycline 100 mg/12 h vs. placebo	Panama	Unclear	Unclear	Unclear	29
Watt G (1988) [29]	RCT	MAT	7 days	Penicillin 1.5 million units/6 h vs. placebo	Philippines	Male and female	Children and adults	Late	42
Edwards CN [30] (1988)	RCT	MAT, ELISA, or *Leptospira* cultures	5 days	Penicillin 2 million units/6 h vs. placebo	West Indies	Unclear	Unclear	Icteric	79
Marotto PC [31] (1997)	RS	MAT, plasma specific, PCR	Unclear	Penicillin 100,000 units/kg of body weight/day or ampicillin 100 mg/kg of body weight/day vs. untreated	Brazil	Male and female	Children	ARF and icteric	43
Daher EF (2000) [32]	RCT	ELISA	8 days	Penicillin 6 million units/day vs. no antibiotic	Brazil	Male and female	Adults	ARF and icteric	35
Costa E (2003) [33]	RCT	The macroscopic slide test, MAT, blood culture, epidemiological findings	7 days	Penicillin 6 million units/day (1 million units/4 h) vs*.* no antibiotic	Brazil	Male and female	Children and adults	Late	253
Panaphut T (2003) [34]	RCT	Serologically proven IgM specific	7 days	Ceftriaxone 1 g/day vs. penicillin 1.5 million units/6 h	Thailand	Male and female	Children and adults	Severe	173
Suputtamongkol Y (2004) [35]	RCT	MAT, IFAT, MCAT	7 days	Penicillin 1.5 million units/6 h vs*.* cefotaxime 1 g/6 h vs. doxycycline 200 mg first dose followed by 100 mg/12 h	Thailand	Male and female	Children and adults	Severe	256
Phimda K (2007) [36]	RCT	MAT	7 days/2 days	Doxycycline 200 mg the first dose followed by 100 mg/12 h vs. azithromycin 1 g initially followed by 500 mg/day	Thailand	Male and female	Children and adults	Late	80
Daher EF (2012) [37]	RS	MAT	Unclear	Penicillin 6 million units/day vs. placebo	Brazil	Male and female	Unclear	ARF	287

*RCT* randomized controlled trial, *CT* controlled trial, *RS* retrospective study, *MAT* microscopic agglutination test, *ELISA* enzyme-linked immunosorbent assay, *PCR* polymerase chain reaction, *IFAT* immunofluorescent antibody test, *ARF* acute renal failure

### Defervescence time

**Fig. 2:**
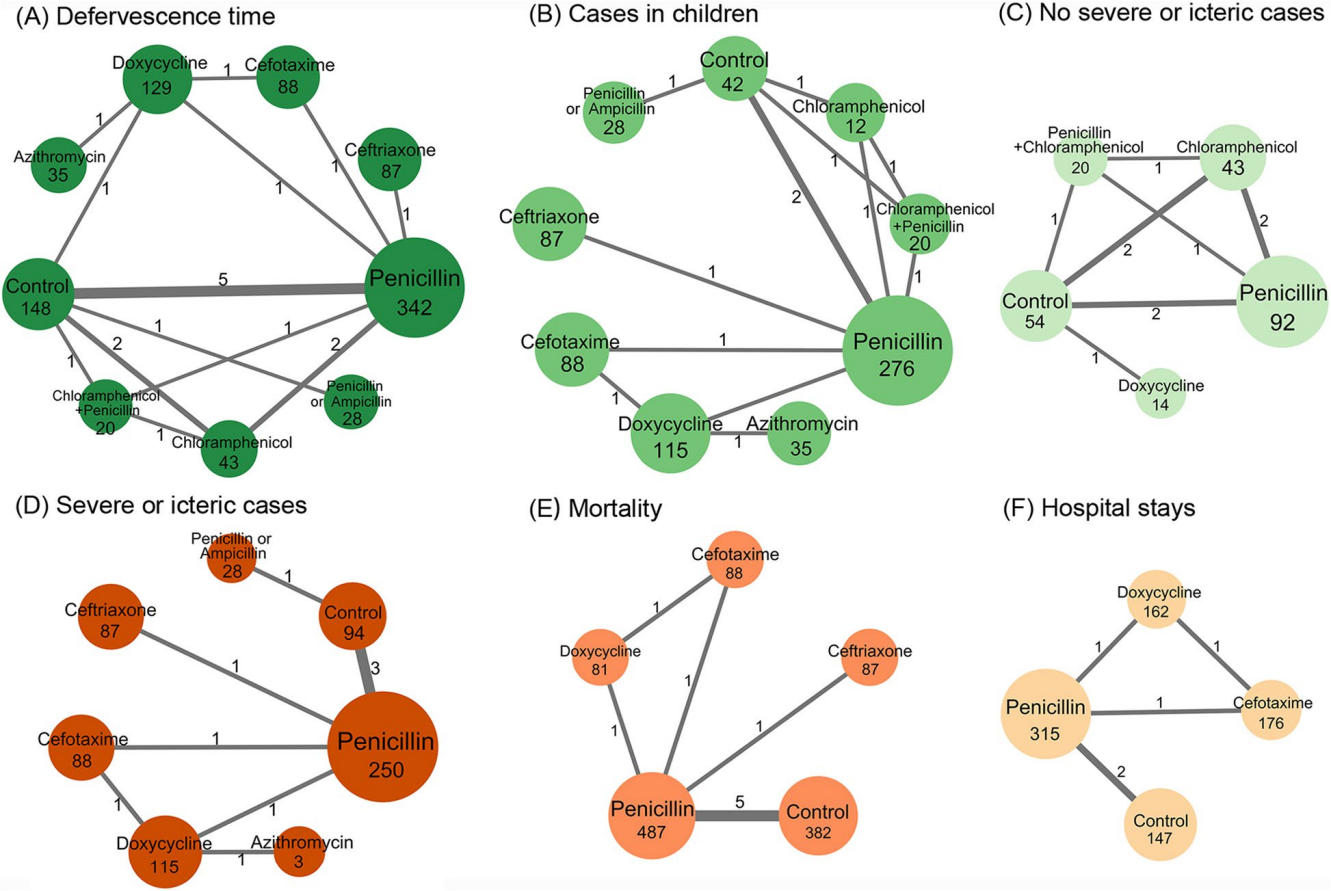
Network plots of available direct comparisons. Each node (solid circle) represents only one type of antibiotic delivery. The size of the nodes is proportional to the number of participants (sample size) involving the specific treatment intervention. The solid lines link treatments being directly compared (the thickness is proportional to the number of trials)

**Fig. 3:**
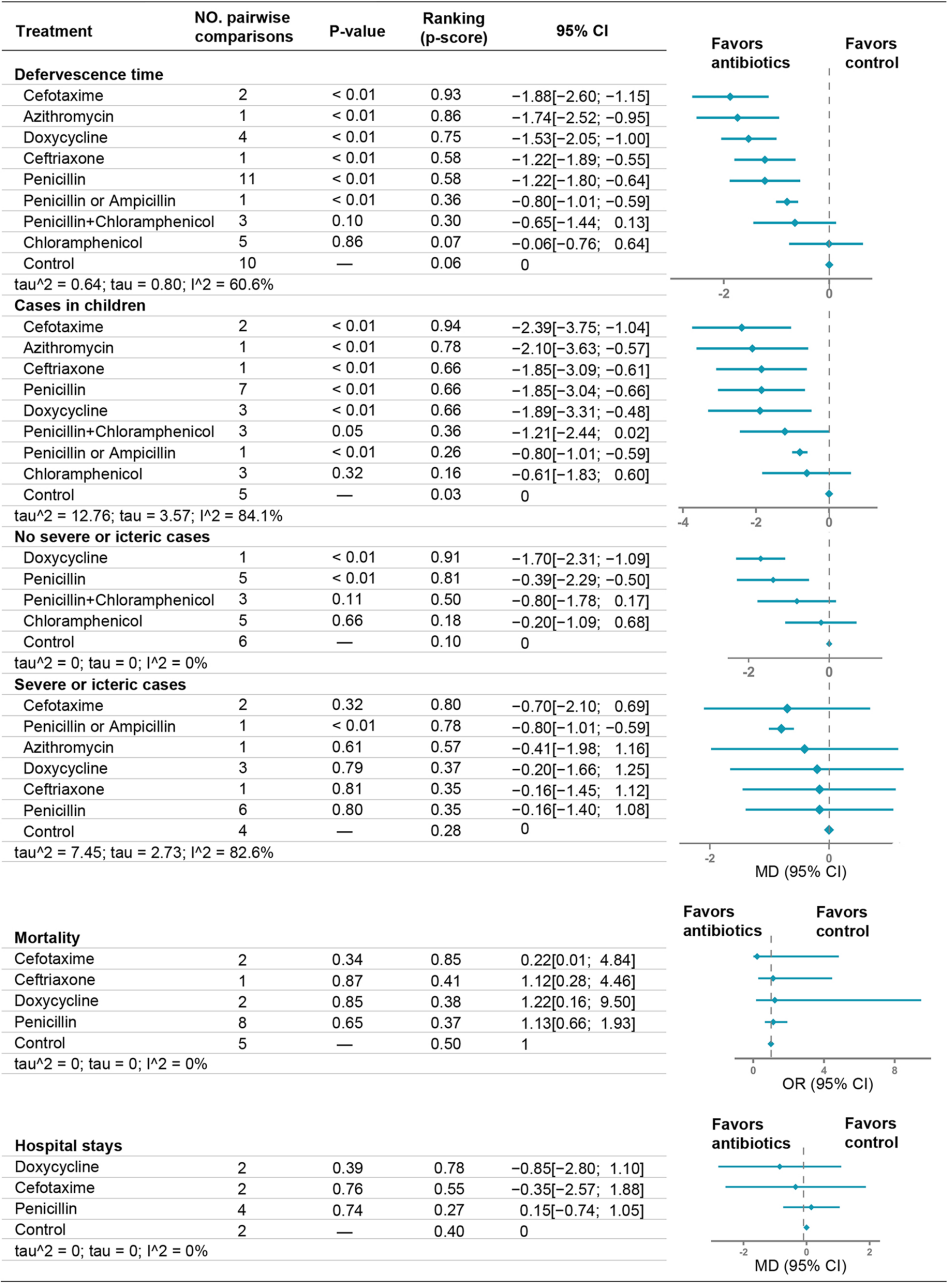
Treatment network meta-analysis and ranking. Other vs*.* control, “other” refers to antibiotics, “control” contains placebo, no antibiotics, ascorbic acid, and no treatment; Tau^2^/tau, quantifying heterogeneity; *I*^2^, quantifying inconsistency; MD, mean deviation; OR, odds ratio. **A** Comparisons of defervescence time for antibiotics and control. The subgroups are cases in children, patients without jaundice or severe disease, and patients with jaundice or severe disease. Cases in children include case studies of children. **B** Comparisons of mortality for antibiotics and controls. **C** Comparisons of hospital stays for antibiotics and controls

Effectiveness was demonstrated on the basis of the defervescence time [25, 26, 28–32, 34–36], mortality [29, 30, 32, 34, 35, 37], and hospital stays [32, 33, 35]. We assessed the effectiveness of eight treatment regimens by measuring their defervescence time (Fig. 2A). We found six interventions that improved the defervescence time including cefotaxime (MD, − 1.88; 95% CI, − 2.60 to − 1.15), azithromycin (MD, − 1.74; 95% CI, − 2.52 to − 0.95), doxycycline (MD, − 1.53; 95% CI, − 2.05 to − 1.00), ceftriaxone (MD, − 1.22; 95% CI, − 1.89 to − 0.55), penicillin (MD, − 1.22; 95% CI, − 1.80 to − 0.64), and penicillin or ampicillin (MD, − 0.08; 95% CI, − 1.01 to − 0.59) (Fig. 3A). By contrast, penicillin + chloramphenicol (MD, − 0.65; 95% CI, − 1.44 to 0.13) and chloramphenicol (MD, − 0.06; 95% CI, − 0.76 to 0.64) had no effect on the defervescence time. We also analyzed the defervescence time in subgroups according to whether there were cases involving children and whether there were cases of severe jaundice. Figure 2B, C shows the corresponding antibiotics, and Fig. 3A shows the antibiotic efficacies. Grouping patients with severe disease and jaundice resulted in a decrease in heterogeneity (*I*^2^ = 0%), compared to the previous value (*I*^2^ = 60.6%; Fig. 3A). This suggests that some of the heterogeneity may originate from seriously ill patients.

### Mortality and hospital stays

Mortality and hospital stays were measured in association with four and three antibiotics, respectively (Fig. 2E, F). We observed similar mortality and hospital stays for all antibiotic and control groups (Fig. 3A), which no heterogeneity was found between the two measures.

### Incidence of adverse reactions

Five studies [28, 30, 35–37] reported adverse reactions after antibiotic treatment for leptospirosis, including three antibiotics (penicillin, doxycycline, and azithromycin). Adverse reactions were mainly Jarisch–Herxheimer reactions for penicillin (2.6%), rashes, headaches, and digestive reactions (nausea, vomiting, diarrhea, abdominal pain, etc.). The rash incidences were 1.1% for penicillin, 0.7% for doxycycline, and 2.0% for azithromycin. We observed the highest incidence of adverse reactions in the digestive system, with a 15.2% of doxycycline users and 6.6% of azithromycin users experiencing vomiting (Table 2).

**Table 2:** Adverse effects of antibiotic therapy

Drug	Adverse effects	Event	Number of patients	Ratio (%)
Penicillin	Jarisch–Herxheimer reaction [25]	1	38	2.6%
	Rash [30]	1	87	1.1%
	Asthenia [32]	70	112	62.5%
	Dark urine [32]	66	112	58.9%
	Abdominal pain [32]	71	112	63.4%
	Epigastric pain [32]	27	112	24.1%
	Hypotension [32]	22	112	19.6%
	Malaise [32]	14	112	12.5%
	Nausea [32]	40	112	35.7%
	Oliguria [32]	42	112	37.5%
	Pallor [32]	52	112	46.4%
	Conjunctival suffusion [32]	25	112	22.3%
	Dizziness [32]	23	112	20.5%
	Cough [32]	41	112	36.9%
Doxycycline	Nausea [31]	3	145	2.1%
	Vomiting [31]	22	145	15.2%
	Nausea and vomiting [31]	10	145	6.9%
	Diarrhea [31]	1	145	0.7%
	Abdominal pain [31]	1	145	0.7%
	Rash [31]	1	145	0.7%
	Dizziness [31]	2	145	1.4%
	Adverse effects or Jarisch–Herxheimer reaction [23]	0	14	0%
Azithromycin	Nausea [31]	1	151	0.7%
	Vomiting [31]	10	151	6.6%
	Nausea and vomiting [31]	1	151	0.7%
	Diarrhea [31]	1	151	0.7%
	Rash [31]	3	151	2.0%

### Ranking

We conducted pairwise comparisons of drugs to establish a ranking of their contribution to defervescence time, mortality, and hospital stays. As shown in Fig. 3, the *p* score decreased in order of efficacy, as in cefotaxime (0.93) > azithromycin (0.86) > doxycycline (0.75) > ceftriaxone (0.58) > penicillin (0.58) > penicillin or ampicillin (0.36). We observed similar mortality and hospital stays with controls and, therefore, do not describe these indicators.

### Inconsistency and publication bias

**Fig. 4:**
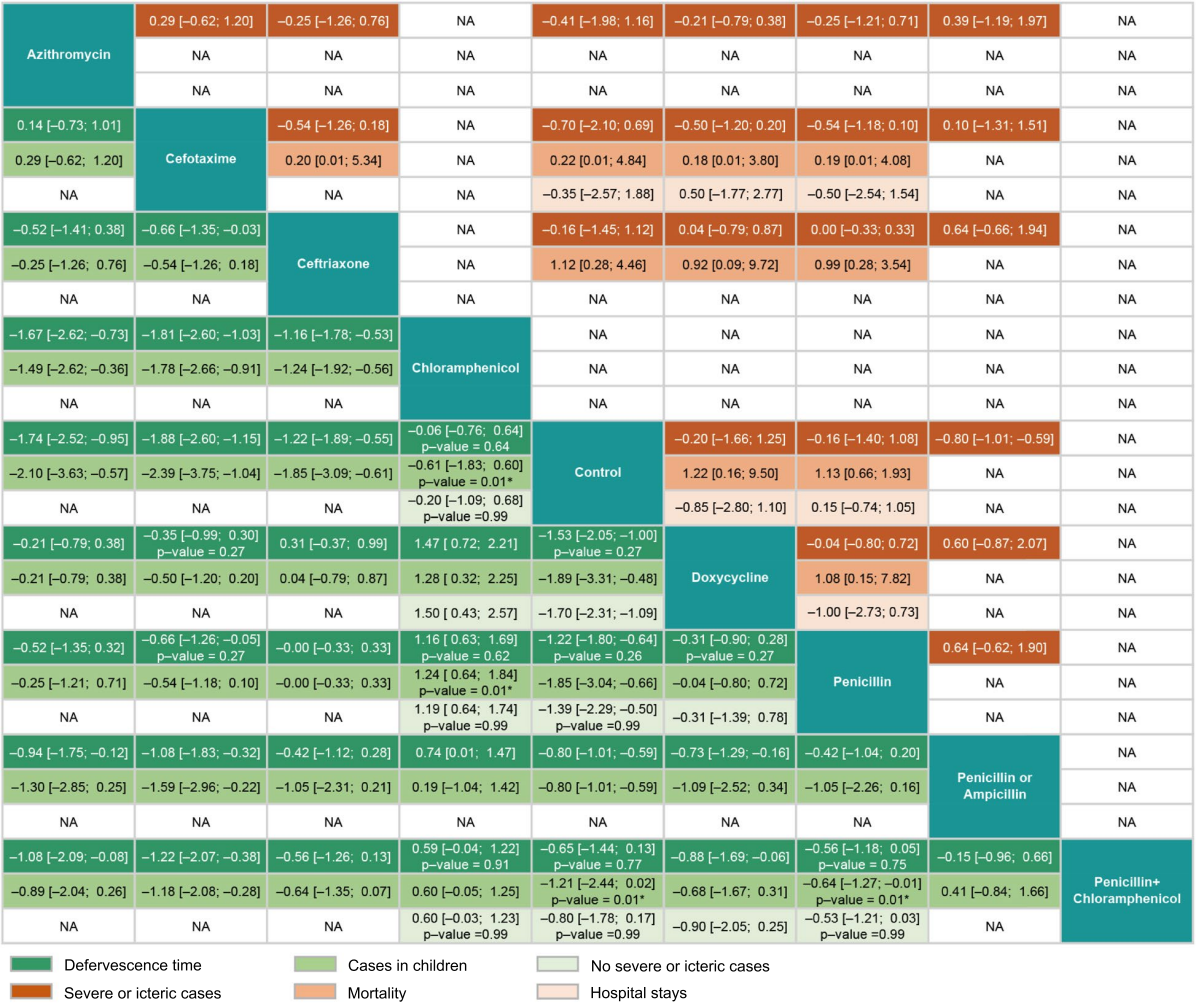
Pairwise comparisons of defervescence time, mortality, and hospital stays for antibiotics and control. Estimated treatment effect (MD) derived from direct and indirect evidence (95% confidence interval); *p*-value, test for disagreement (direct versus indirect); *p < 0.05

The results of the evaluation of the inconsistency of the defervescence time across all comparisons are presented in Fig. 4. The analysis revealed statistically significant differences between the four groups in children: chloramphenicol vs*.* control (*p* < 0.01), chloramphenicol vs. penicillin (*p* < 0.01), penicillin + chloramphenicol vs. control (*p* < 0.01), and penicillin + chloramphenicol vs*.* penicillin (*p* < 0.01). These groups differed in their comparative analysis of direct evidence vs*.* indirect evidence (eFigure 1).

We found no evidence of publication bias in the defervescence time, as indicated by Begg’s funnel plot and Egger’s test results (*p* = 0.65). However, due to the limited number of studies, we could not apply Egger’s test to the other two indicators (mortality and hospital stays). See eFigure 2 for further details.

## Discussion

Broad-spectrum antibiotics to cover bacteria are usually required while a definite diagnosis of leptospirosis is pending [34]. However, no exact guidelines on the type of antibiotic that should be prescribed exist, and the effectiveness of this approach has yet to be demonstrated. Improper use of antibiotics may lead to the emergence of resistant strains, adverse side effects, and increased medical costs [8]. Thus, this meta-study evaluating the effectiveness of antibiotics for *Leptospira* was needed. We aimed to identify the best antibiotic option to treat leptospirosis through a comprehensive meta-analysis, searching the literature in English using various related terms.

Despite the worldwide distribution of leptospirosis, only a small number of RCTs evaluating treatments have been performed [27–30, 32–35]. Unfortunately, the existing findings are conflicting, with some studies concluding that antibiotic treatment for spirochetes is beneficial [25, 27–29, 31, 34–37], while others have indicated that it is not effective [26, 30, 32, 33]. Some studies have recommended penicillin as the standard antibacterial drug for the treatment of moderate to severe leptospirosis [25, 29], but others found poor efficacy for patients with advanced severe leptospirosis [30, 32, 33]. Cefotaxime and ceftriaxone are third-generation cephalosporins with potential efficacy against leptospirosis. They can effectively inhibit the growth of *Leptospira* and shorten the duration of leptospirosis [34, 35]. Cephalosporins are preferred over penicillin because they are simpler to administer (ceftriaxone once a day given either intravenously or intramuscularly vs. penicillin four times per day given only intravenously) and more readily available. Also, in many cases of penicillin allergy, it is possible to safely administer a cephalosporin [38, 39]. Another favored antibiotic is doxycycline, which shortens the duration of the disease and has favorable effects on fever, malaise, headache, and myalgia [28, 36]. Doxycycline, which has been recommended and used widely for the prophylaxis and treatment of leptospirosis of mild severity [38, 40, 41], is also active against *Rickettsia* organisms. Azithromycin, although expensive, may be a good alternative for the treatment of leptospirosis, especially when drug resistance is suspected [36]. Clinical studies suggest that oxytetracycline may have good efficacy in the treatment of leptospirosis and that chloramphenicol has no efficacy [26, 27]. The tissue localization stages of leptospirosis have been identified and organ damage has been observed, but antibiotics may be of little value in regulating the disease process [30]. We found that the studies reporting limited significance for antibiotic treatment of leptospirosis were conducted in patients with severe, jaundiced, and acute renal failure leptospirosis, and we cannot exclude that our results were affected by this selection of patients [26, 30, 32, 33].

Fever is one of the most common symptoms of leptospirosis, and we evaluated the time to fever reduction with antibiotics. Six types of medications (cefotaxime, azithromycin, doxycycline, ceftriaxone, penicillin, and penicillin or ampicillin group) significantly reduced the defervescence time. However, the antibiotics were not effective in reducing the mortality and hospital stay lengths. We speculate that the smaller number of included studies and larger number of included severe leptospirosis cases have an impact on the evaluation of mortality and hospital stay lengths. The main adverse reactions after antibiotic use included Jarisch–Herxheimer reactions, rash, headache, and digestive reactions (nausea, vomiting, diarrhea, and abdominal pain). We found that most of the included articles discussed severe or jaundiced leptospirosis, so we performed a subgroup study on the defervescence time for these patients. Antibiotics were ineffective for treating severe or jaundiced leptospirosis, with the exception of the penicillin or ampicillin group, which showed some efficacy. Severe or advanced disease reflects the second stage of leptospirosis, which is largely considered an immune-mediated event [42, 43]. The use of antibiotic potency at this stage is controversial. The limited number of studies included in our analysis prevents us from drawing definitive conclusions regarding the efficacy of antibiotics in severe/late leptospirosis.

Evaluating antibiotic therapies for leptospirosis is difficult due to the wide range of severity and complications associated with the disease. Mild clinical symptoms may not require antibiotic treatment and may resolve on their own [44]. However, as the disease can potentially evolve to a more severe stage, with life-threatening complications, we recommend that the patient be treated with antibiotics in time after diagnosis. Although antibiotics have no meaningful impact on the lethality of the infection, they can accelerate the defervescence, thereby alleviating the patient’s discomfort and buying time for treatment. Therefore, we recommend the use of cephalosporins, doxycycline, or penicillin for the treatment of leptospirosis. Azithromycin is a potential drug-resistance alternative.

The management of antibiotic therapy for leptospirosis is fraught with problems: (i) the lack of experimental and clinical data, as well as the lack of understanding of the pathophysiology of the disease, has hindered progress in the field of antibiotic treatment of leptospirosis. (ii) The leptospirosis diagnoses are commonly delayed, and some experts recommend that medication should be administered as soon as leptospirosis is suspected [44, 45]. (iii) The use of antibiotics in severe or advanced leptospirosis is controversial, and the terms “severe” and “late” have been used interchangeably with differing definitions. This is probably due to the notion that protracted clinical disease is de facto severe or prone to progressing to severe complications [44–46] and has implications for treatment. Prompt diagnoses and initiation of appropriate therapy are important for managing leptospirosis.

The sources of infection included in this paper were from Australia, Malaya, Malaysia, Panama, the Philippines, the West Indies, Brazil, and Thailand. Most of these places are tropical developing countries with a high incidence of leptospirosis. High temperatures, stagnant water, and poor sanitation all contribute to the high incidence of leptospirosis in the tropics. There are more *Leptospira* serotypes in the tropics and there is no literature on the relationship of these serotypes to treatment and drug resistance. Clinical trials of antibiotic treatment for leptospirosis are few and old, and it is difficult to discern differences in antibiotic treatment and resistance in different regions from the available data. It is hoped that more studies will supplement these deficiencies at a later stage.

This study has limitations. First, while we only included clinical control trials, some of these were not randomized control trials, which may lead to variable results. We did a bias analysis and did not find any bias in the results due to non-RCT data. Second, we did not have access to high-quality data on all drugs and mortality, which may affect efficacy and safety to some extent. Third, there is insufficient RCT data to produce results on the duration of drug treatment, dosage, etc. Lastly, we did find evidence of inconsistency in the results from our indirect comparison analysis. These findings should be interpreted with caution as the low number of pairwise comparisons suggests that there may be significant differences in drug efficacy from a clinical perspective.

## Conclusions

Although antibiotics have no meaningful impact on the mortality and hospital stays of the leptospirosis infection, they can accelerate the defervescence, thereby alleviating the patient’s discomfort and buying time for treatment. Cephalosporins, doxycycline, penicillin, and azithromycin are recommended for leptospirosis.

### Supplementary Information

** Supplementary Material 1.**  

## Data Availability

The data that support the findings of this study are available from the corresponding author upon reasonable request.

## Abbreviations

RCTs: Randomized clinical trials
CTs: Clinical trials
RSs: Retrospective studies
NMA: Network meta-analysis
PRISMA: Preferred Reporting Items for Systematic Reviews and Meta-Analysis
MAT: Microscopic agglutination test
ELISA: Enzyme-linked immunosorbent assay
PCR: Polymerase chain reaction
ORs: Odds ratios
CIs: Confidence intervals
MDs: Mean deviations
